# Socioeconomic inequality in self-rated health and its determinants: an Oaxaca blinder decomposition in Ilam, West of Iran during 2023

**DOI:** 10.1186/s12913-023-10242-y

**Published:** 2023-11-03

**Authors:** Mohammad Bazyar, Hojatollah Kakaei, Mohsen Jalilian, Amin Mirzaei, Mohammad Ali Mansournia, Reza Pakzad

**Affiliations:** 1 Department of Health Management and Economics, Faculty of Health, Ilam University of Medical Sciences, Ilam, Iran; 2 Department of Occupational Health, Faculty of Health, Ilam University of Medical Sciences, Ilam, Iran; 3 Department of Public Health, Faculty of Health, Ilam University of Medical Sciences, Ilam, Iran; 4 Department of Epidemiology, School of Public Health, Tehran University of Medical Sciences, Tehran, Iran; 5 Health and Environment Research Center, Ilam University of Medical Sciences, Ilam, Iran; 6 Psychosocial Injuries Research Center, Ilam University of medical Sciences, Ilam, Iran; 7 Department of Epidemiology, Faculty of Health, Ilam University of Medical Sciences, Banganjab, Pajouhesh Blvd, Ilam, Iran; 8 Student Research Committee, Ilam University Medical Sciences, Ilam, Iran

**Keywords:** Socioeconomic inequality, Self-rated, Oaxaca Blinder Decomposition, Ilam, Iran

## Abstract

**Aim:**

To determine inequality and decompose it’s in Self-Rated Health (SRH).

**Method:**

This population-based cross-sectional study was undertaken on the entire population of the city of Ilam, Iran, in 2023. Multi-stage stratified cluster random sampling with proportion-to-size approach was used to select the participants. Oaxaca-Blinder decomposition technique was used to show the amount of inequity in SRH and to decompose of the gap of SRH between the poor and the rich group of participants.

**Results:**

1370 persons participated in the study. The 59.38% of participants stated good SRH status and just 8.86% of participants had poor SRH status. The results of the Oaxaca-Blinder decomposition revealed a considerable gap (15.87%) in the poor status of SRH between the rich and the poor. A large proportion (89.66%) of this difference was described by explained portion of the model. The results of decomposition showed that economic status was directly responsible for explaining 27.98% of overall inequality gap between rich and poor people. Moreover, hopelessness to future (32.64%), having an underlying disease (18.34%) and difference in the education level (10.71%) were associated with an increase in inequality disfavoring the poor.

**Conclusion:**

For people suffering from underlying disease, it is suggested to devise policies to improve access to/and remove healthcare utilization barriers. To address hopelessness to future, it is recommended to carry out further studies to reveal factors which affect it in more details. This can help policy makers to formulate more realistic and evidence-informed policies on order to lessen the current socioeconomic inequity in SRH.

**Supplementary Information:**

The online version contains supplementary material available at 10.1186/s12913-023-10242-y.

## Background

Socioeconomic inequalities in health are a great public health challenge in both developed and developing countries and have become an increasing research interest in fields of epidemiology and health economics [1–3]. For this reason, over the last decades, besides working on improving health for all groups of population; policymakers, health experts, and health officials have been struggling to reduce or remove disparities in health which exist among people in different groups of population [1–6].

Over the last years, different approaches have been devised focusing on the causes of health inequities between various social and economic layers of the societies. For example, socioeconomic status (SES) was introduced by the World Health Organization (WHO) as an appropriate approach to categorize people into hierarchical groups to extract health inequities existing among them. Other factors proposed by WHO to group people socially and economically for health equity purposes are place of residence, race/ethnicity, occupation, gender, religion, education, and social capital or resources [7]. SES measures is a relative (not absolute) approach in which the socioeconomic position of individuals’ and their social groups are defined relatively in comparison with other people around them living in their society [4]. SES is an aggregate concept that merges different social and economic items into a single compound variable to determine the relative position of each person within the society and the diverse pathways by which SES may affect health [8].

The strong relationship between SES and inequity in health have been proven already by the many studies in different aspect including access to various health care services, different mental and physical health disorders, and also enjoying better health status [9–13]. Another health component that has been increasingly weighed in different layers of SES to measure equity in health, is self-rated health (SRH) (or self-assessed health, or self-perceived health). SRH is one of the key determinants of general health, functionality, and mortality [14]. It is a simple question asking people to evaluate their health status or to compare their health status with the health of age peers in the society [15]. This simple question [16] has been used increasingly in epidemiological, psychological research, clinical settings, health economics studies [17–21], and also in major national and international general population surveys by international organizations and in developed countries [22, 23].

Prior studies on different age groups have shown that people with lower SES are more likely to state poorer self-perceived health [24–26]. For example, in a study was done in Tehran, Iran, in 2008 [27], they showed that sub-optimal health was reported 3.67 times more by the poor in comparison with the rich. The main contributors explaining the socioeconomic inequity were economic status (47%), level of education (29.2%) and age (23%). According to other relevant studies, following socioeconomic variables including educational levels [28], age, number of diseases, perceived family respect, neighborhood relations, economic dependence, residential differences, wealth status the percentage of income spent on rent [29], gender differences, body dissatisfaction and weight perceptions, parent relation, family income, physical exercise, and school achievement [30, 31] have been proven to affect self-rated health.

Despite the usefulness and increasingly using of SRH as the main outcome in health equity studies, few studies have investigated the impact of socioeconomic status in SRH on Iran which have been mainly based on school setting national survey [27, 32, 33]. Previous health equity studies in Iran mainly have investigated access to different health care services or suffering from different kinds of diseases [27, 34, 35]. More studies about inequity in SRH in other region of the country focusing on new factors are required to prepare more thorough and comprehensive picture about the amount of socioeconomic inequity in SRH and its main contributors. Findings from different regions with different socioeconomic situation provide deeper understanding of the subject and the similarities and differences among findings of the studies can present more reliable and informative data for informed health policy making. This in turn can help decision makers to adapt both general and region-specified policies to combat with available health inequities. To do so, we decided to investigate socioeconomic inequity in self-rated health in Ilam province as it is known as one of the deprived regions of Iran where most of economic and development indicators indicate that it is among the worst provinces of Iran [36, 37] suffering from many undesirable economic issues. As one of the 31 provinces of Iran, Ilam province is the least populated province, with 580 158 people according to the 2016 census. To the authors’ best knowledge, this is the first study aiming to measure inequity in health using the Blinder-Oaxaca method of self-reported health in the west of Iran. So we aim to determine to what extent inequalities in SRH are affected by difference in SES and use Oaxaca Blinder decomposition to identify the strongest predictors when taking all SES indicators into account.

## Method

**Fig. 1:**
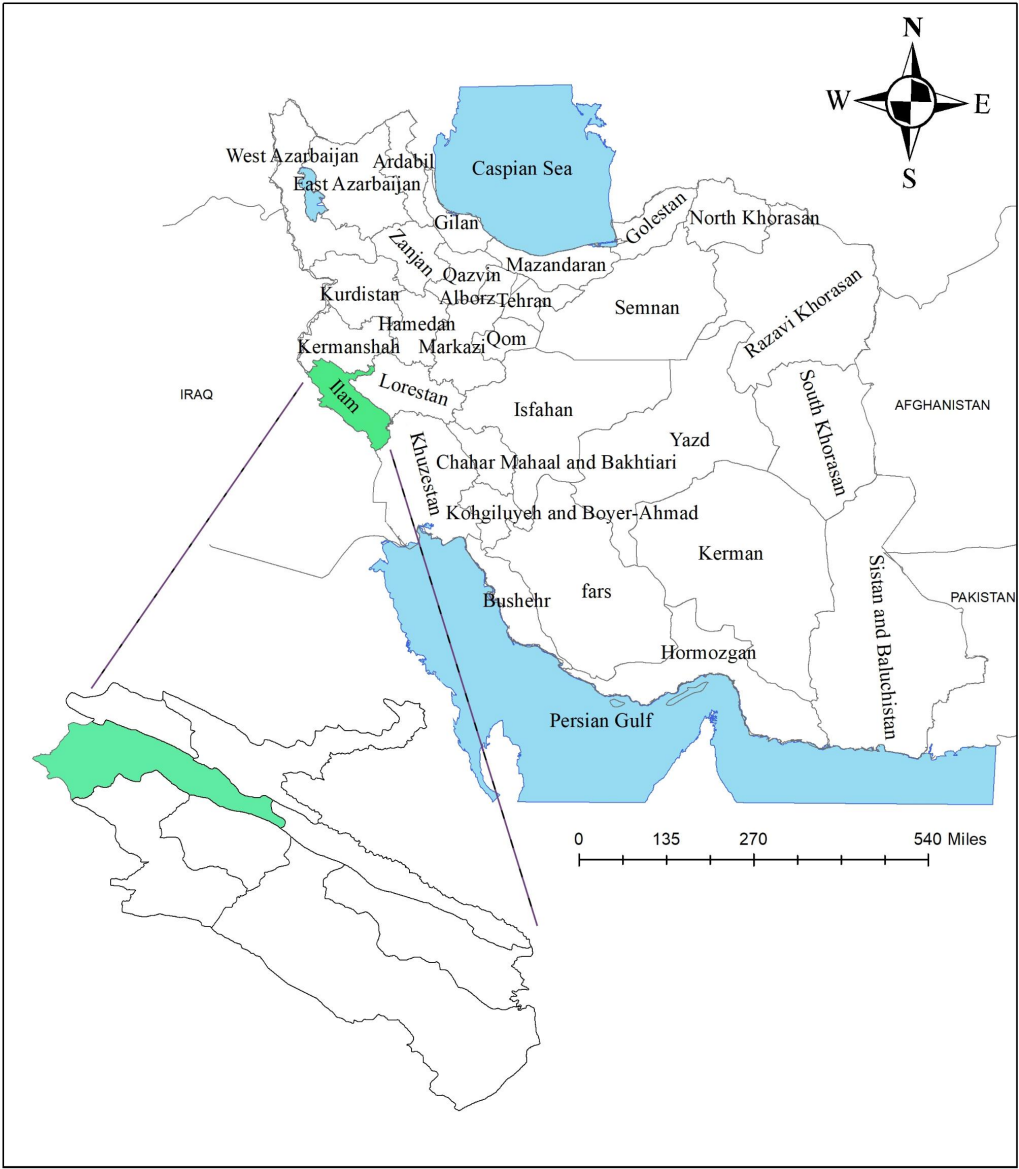
Geographical location of Ilam city

This cross-sectional survey was conducted in city of Ilam, located in the west of Iran, with a population of 201 thousand people (Fig. 1). According to Maharlouei et al.‘s study [33], considering a rate of good-SRH as 47.3%, a confidence level 95% and the precision = 0.05, based on the following formula, the sample size was estimated of 381.

$${\rm{n}}\,{\rm{ = }}\,{\rm{[}}{1.96^ \wedge }2\, \times \,(0.54\, \times \,0.46)]/\,{0.05^ \wedge }2\, = \,381$$

According to the type of sampling, considering the design effect equal to 2.5, a 10% attrition rate and 20% for missing data rate, finally 1230 people was considered as the minimum final sample size. Sampling was done from January 5, 2023, to March 16, 2023. The study used multi-stage stratified cluster random sampling with a proportion-to-size approach to select participants. The city of Ilam was divided into 10 divisions based on health centers, and the population covered by each region was obtained. Sample size was calculated based on population, and cluster sampling was used with a sample size of 20 people per cluster. Sampling teams were dispatched to selected clusters, and households were selected in a counterclockwise manner by inviting individuals over the age of 15 to participate after explaining the study objectives and ensuring data confidentiality and anonymity.

Data collection was completed using face-to-face structured interviews undertaken by trained questioners (Interview guide is provided as supplementary file 1). In following and after explaining the study objectives, while obtaining informed consent, the study information was collected by questionnaire. The data collected included demographic variables (age, gender, race, education, household dimensions, occupation and insurance coverage), economic status (asset-based approach) and other information (co-morbidities, history of mental disorder, history of death of family members, history of job loss and people’s hope for the future). In this study, we used a single question about hope for the future: “Based on the overall situation in the country and society, do you have any hope for the future?“

In this study, SRH was the dependent variable and was indicated by the question ‘In general, how would you rate your health?’ using the Likert-scale of very good, good, fair, poor and very poor. To assess the clear differentiation among participants, we combined the responses of very good and good into one category of ‘good SRH status’ and combined the responses of poor and very poor into a second category of ‘poor SRH status’. The validity and reliability of this tool was previously confirmed by Maharlouei et al. [33]

### Statistical analysis

The economic status of participants was determined by collecting data on 15 household assets (including cars, motorcycles, refrigerators, washing machines, macro-waves, laptops, vacuums, dish washers, access to the internet, LCD TVs, DVD players, going to restaurants, traveling, having a house, and steam irons) and generating a wealth index using principle component analysis. The index ranges from infinitely negative to infinitely positive, with lower values indicating poor status and higher values indicating rich status.

The concentration index was used to measure inequality in SRH over the distribution of the wealth index. Also, a concentration curve was used to plot the cumulative proportion of SRH against the cumulative proportion of the population ranked by wealth index. [27].

Then, the participants were divided to three groups (poor, middle and rich), and the Oaxaca-blinder decomposition method was used to decompose the gap between the poor and rich to its determinants [10, 38–40]. In this method, the following formula is applied to model the mean outcome variable according to the determinant variables in each economic group:

1 $${\overline{\text{Y}}}^{\text{p}\text{o}\text{o}\text{r}}= {{\beta }\text{x}}^{\text{p}\text{o}\text{o}\text{r}}+ {{\epsilon }}^{\text{p}\text{o}\text{o}\text{r}}$$

2 $$(2) {\overline{\text{Y}}}^{\text{r}\text{i}\text{c}\text{h}}= {{\beta }\text{x}}^{\text{r}\text{i}\text{c}\text{h}}+ {{\epsilon }}^{\text{r}\text{i}\text{c}\text{h}}$$

Where $\overline{\text{Y}}$ is the mean outcome variable, β is the model coefficient including the intercept, ${\epsilon }$ is the model error, and x is the explanatory variable.

The gap between the two economic groups can be formulated as:

3 $${\overline{\text{Y}}}^{\text{p}\text{o}\text{o}\text{r}}-{\overline{\text{Y}}}^{\text{r}\text{i}\text{c}\text{h}} =\left({\overline{\text{x}}}^{\text{r}\text{i}\text{c}\text{h}}-{\overline{\text{x}}}^{\text{p}\text{o}\text{o}\text{r}} \right) {{\beta }}^{\text{p}\text{o}\text{o}\text{r}}+ \left({{\beta }}^{\text{r}\text{i}\text{c}\text{h}}-{{\beta }}^{\text{p}\text{o}\text{o}\text{r}} \right) {\overline{\text{x}}}^{\text{r}\text{i}\text{c}\text{h}}$$

4 $${\overline{\text{Y}}}^{\text{r}\text{i}\text{c}\text{h}}-{\overline{\text{Y}}}^{\text{p}\text{o}\text{o}\text{r}} =\left({\overline{\text{x}}}^{\text{r}\text{i}\text{c}\text{h}}-{\overline{\text{x}}}^{\text{p}\text{o}\text{o}\text{r}} \right) {{\beta }}^{\text{r}\text{i}\text{c}\text{h}}+ \left({{\beta }}^{\text{r}\text{i}\text{c}\text{h}}-{{\beta }}^{\text{p}\text{o}\text{o}\text{r}} \right) {\overline{\text{x}}}^{\text{p}\text{o}\text{o}\text{r}}$$

The gap between the two economic group is divided to two portions: 1- explained portion, which is the first part of the right hand side of the above formulas and is due to differences in the mean values of the variables between the two groups, and 2- unexplained portion, which is the second part of the right hand side of the above formulas and is due to differences in the coefficients of these variables [41, 42]. To analyze the binary outcome, the method developed by Yun for non-linear outcomes was utilized [43]. To decompose the gap, the role of the determinant variables in the explained and unexplained portions was evaluated.

For model building of oaxaca-blinder decomposition, three steps were applied. At the first step, the association between SRH with determinant variables (including gender, economic status, race, marital status, education, job, underlying diseases, insurance, use of health care service, visited by a doctor in the last year, smoking, alcohol, hookah, losing of family members, mental disorder history, hope to future, job losing history, BMI, family size, age) was evaluated using simple logistic regression. Then a multiple logistic regression model was developed using only variables that had a significant effect in the first step. Lastly, variables that had a significant effect in the second step, considered eligible for inclusion in the decomposition model.

It should be emphasized that economic status was also included in the decomposition model to investigate its direct effects on economic inequality besides its indirect effects. The Oaxaca command in the Stata software version 11 was used to analyze inequality [44] and the cluster sampling effect was considered in calculating the confidence intervals. P values less than 0.05 were considered significant.

## Result

The data of 1370 participants was analyzed. The distribution of demographic and other variables was shown in (Table 1). The age mean (SD) of the participants was 40.45 (15.42). 50.30% (47.63 to 52.96) of participants were female, 94.39% (93.16 to 95.61) were Kurds’ ethnicity, 61.96% (59.38 to 64.55) were married, 35.6% (33.04 to 38.15) had an education level higher than that of a bachelor, and 31.39% (28.91 to 33.86) were classified as a high economic group. 59.38% (56.76 to 62.01) of participants stated good SRH status, and just 8.86% (7.35 to 10.38) of participants had poor SRH status.

**Table 1:** distribution of demographic variables in study population

Variables			Number	Effect size (95% CI)
Percent	Gender	Male	680	49.70 (47.04 to 52.37)
		Female	690	50.30 (47.63 to 52.96)
	SRH	Good	814	59.38 (56.76 to 62.01)
		Fair	435	31.76 (29.27 to 34.24)
		Poor	121	8.86 (7.35 to 10.38)
	Economic status	Low	511	37.22 (34.64 to 39.80)
		Middle	430	31.39 (28.91 to 33.86)
		High	429	31.39 (28.91 to 33.86)
	Ethnicity	Kurd	1292	94.39 (93.16 to 95.61)
		Other	78	5.61 (4.39 to 6.84)
	Marital status	Married	840	61.96 (59.38 to 64.55)
		Single	454	33.53 (31.01 to 36.05)
		Divorce or widow	61	4.51 (3.40 to 5.61)
	Education	< Diploma	422	31.02 (28.55 to 33.49)
		Diploma and Associate Degree	457	33.38 (30.87 to 35.90)
		≥ Bachelor	491	35.6 (33.04 to 38.15)
	Job	Student	193	14.18 (12.32 to 16.04)
		Employed	216	15.58 (13.65 to 17.52)
		Retrieved	153	11.15 (9.47 to 12.83)
		Housekeeper or unemployed	808	59.08 (56.46 to 61.71)
	underlying diseases (Yes)		479	34.86 (32.32 to 37.40)
	Insurance (Covered)		1039	76.07 (73.80 to 78.35)
	Use of health care service (Yes)		770	56.35 (53.71 to 59.01)
	Visited by a doctor in the last year (Yes)		1041	76.14 (73.87 to 78.42)
	Smoking (Yes)		134	9.78 (8.21 to 11.36)
	Alcohol (Yes)		37	2.7 (1.84 to 3.56)
	Hookah (Yes)		120	8.76 (7.26 to 10.26)
	Losing of family members (Yes)		664	48.47 (45.82 to 51.12)
	Mental disorder History (Yes)		113	8.25 (6.79 to 9.71)
	Hope to future (Yes)		803	58.61 (56.01 to 61.22)
	Job losing History (Yes)		286	20.88 (18.72 to 23.03)
Mean ± SD	BMI (Kg/M^2^)		1370	25.80 ± 4.19
	family size (Number)		1370	4.24 ± 1.58
	Age (Yrs. old)		1370	40.45 ± 15.42

*Present as Mean ± SD; SES: socioeconomic status; BMI: Body Mass Index; SRH: Self-Rated Health; SD: Standard Deviation

As mentioned in the method, for the purpose of developing a multiple logistic regression model, only variables with a significant effect were eligible to be included in multiple logistic regression. The results are shown in (Table 2). In comparison to poor economic status, the odds of poor status for SRH in middle and rich groups were 0.44 (CI 95%: 0.24 to 0.81) and 0.61 (CI 95%: 0.32 to 0.89), respectively. Hopelessness to future [OR: 12.89 (CI 95%: 7.40 to 22.46)] and having an underlying disease [OR: 5.58 (CI 95%: 3.27 to 9.50)] were positively associated with the poor status of SRH. Also, having an equal or higher bachelor’s educational level was negatively associated with the poor status of SRH [OR: 0.52 (CI 95%: 0.27 to 0.98)]. Other included determinants, including smoking, marital status, losing family members, history of mental health disorders, or losing a job, did not show a statistically significant association with SRH.

**Table 2:** Association between poor status of SRH with determinant variables using multiple logistic regression

Variables		SRH		VIF
		OR (95% CI)	p-value	
**Economic status**	Poor	**Reference**		2.10
	Middle	0.44 (0.24 to 0.81)	0.009*	
	Rich	0.61 (0.32 to 0.89)	0.007*	
**Losing of family members**	No	**Reference**		2.44
	Yes	1.49 (0.87 to 2.56)	0.148	
**Mental disorder history**	No	**Reference**		1.12
	Yes	1.65 (0.76 to 3.59)	0.194	
**Hopelessness to future**	No	**Reference**		3.18
	Yes	12.89 (7.40 to 22.46)	< 0.001*	
**Smoking**	No	**Reference**		1.15
	Yes	1.19 (0.57 to 2.49)	0.635	
**Marital status**	Married	**Reference**		1.12
	Single	1.40 (0.73 to 2.69)	0.312	
	Divorce or widow	1.89 (0.73 to 4.90)	0.188	
**Underlying disease**	No	**Reference**		3.10
	Yes	5.58 (3.27 to 9.50)	< 0.001*	
**Insurance**	No	**Reference**		2.62
	Yes	1.67 (0.97 to 2.86)	0.064	
**Education**	< Diploma	**Reference**		1.24
	Diploma & Associate Degree	0.62 (0.35 to 1.12)	0.113	
	≥ Bachelor	0.52 (0.27 to 0.98)	0.046*	
**BMI (kg/M** ^**2**^ **)**		1.01 (0.95 to 1.07)	0.784	3.18
**Age (yrs. old)**		1.01 (0.98 to 1.02)	0.669	1.83

*: Significant at 0.05 level; BMI: Bod Mass Index; SRH: Self-Rated Health, VIF: variance inflation factor

**Fig. 2:**
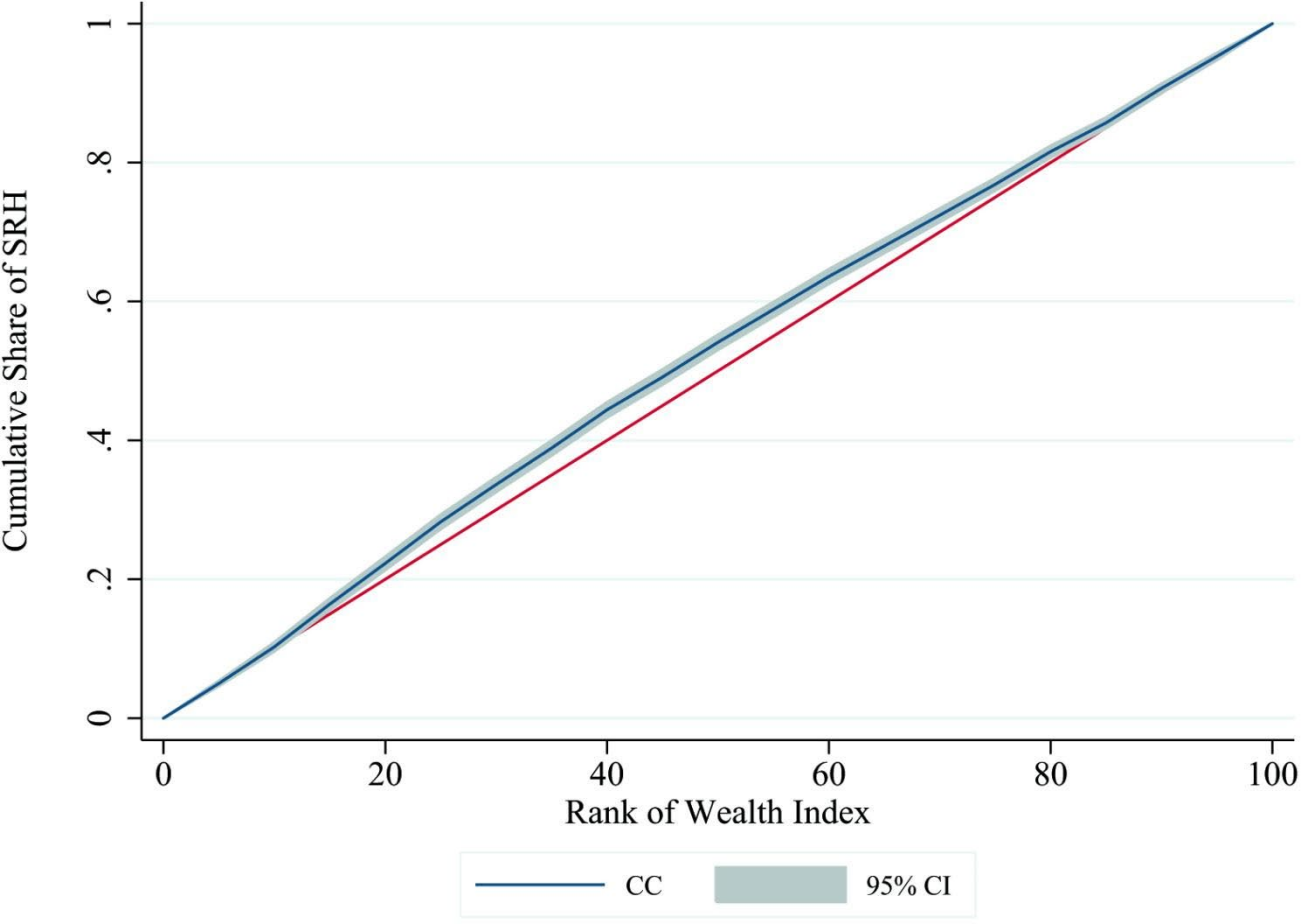
Concentration curve for self-rated health against wealth index rank

Based on the result of the concentration index, there was a pro-poor inequality, so the value of the concentration index was − 0.0454 (p < 0.001). Figure 2 shows the concentration curve. (Table 3) presents the results of the Oaxaca-Blinder decomposition. A significant gap was found in SRH between the rich and poor economic groups, such that the difference in the rate of poor status of SRH between the two groups was 15.87%, dis-favoring the poor (p < 0.001). The explained portion comprised 89.66% (14.23/15.87) of the difference (p < 0.001); in other words, the gap was mostly due to the difference in the mean values of the variables between the rich and poor groups, and only 10.27% (1.63/15.87) of the gap was due to the unexplained portion, whose effect was not significant (p: 0.194).

**Table 3:** result of gap decomposition of poor SRH status in poor and rich groups using Oaxaca-Blinder decomposition

Blinder-Oaxaca decomposition results		Coefficient (95% CI)	p-value		
Prevalence	In poor economic group	23.05 (18.44 to 27.66)	< 0.001		
	in rich economic group	7.19 (4.36 to 10.02)	< 0.001		
	total gap	15.87 (10.45 to 21.28)	< 0.001		
**Explained portion**		**Coefficient (95% CI)**	**p-value**	**% of total gap**	**% of subtotal gap**
Economic status		4.44 (0.16 to 8.73)	0.042	27.98	31.20
Hopelessness to future		5.18 (3.09 to 7.26)	< 0.001	32.64	36.40
Underlying disease		2.91 (1.34 to 4.48)	< 0.001	18.34	20.45
education	< Diploma	1			
	Diploma & Associate Degree	-0.13 (-0.51 to 0.25)	0.497	-0.82	-0.91
	≥ Bachelor	1.83 (0.26 to 3.41)	0.022	11.53	12.86
	Sum	1.70 (0.25 to 3.66)	0.021	10.71	11.95
Subtotal Gap		14.23 (9.37 to 19.1)	< 0.001	89.66	100
**Unexplained**		**Coefficient (95% CI)**	**p-value**	**% of total gap**	**% of subtotal gap**
Economic status		-11.09 (-20.43 to -1.75)	0.020	-69.88	-680.37
Hopelessness to future		4.26 (0 to 8.52)	0.050	26.84	261.35
Underlying disease		5.63 (1.84 to 9.42)	0.004	35.48	345.40
education	< Diploma	1			
	Diploma & Associate Degree	-1.27 (-9.39 to 6.85)	0.526	-8.00	-77.91
	≥ Bachelor	-1.74 (-4.79 to 1.31)	0.264	-10.96	-106.75
	Sum	-3.01 (-14.18 to 2.62)	0.687	-18.97	-184.66
Constant		5.85 (-8.32 to 20.02)	0.419	36.86	358.90
Subtotal Gap		1.63 (-0.83 to 4.1)	0.194	10.27	100

The results of inequality decomposition showed that economic status was a significant determinant of inequality, and 27.98% of the overall gap was due to the direct effect of economic status (Coefficient: 13.24; p < 0.001). Economic status comprised 31.20% of the explained portion. Moreover, hopelessness to future was also associated with an increase in inequality disfavoring the poor (coefficient: 5.18; p < 0.001) so that 32.64% of the overall gap was due to a higher rate of hopelessness in poor people. About 18.34% of the overall gap was explained by having an underlying disease. In other words, a higher rate of having an underlying disease in poor people leads to an increase in inequality (coefficient: 2.91; p < 0.001). The difference in education level between the two groups comprised 10.71% of the overall gap.

**Fig. 3:**
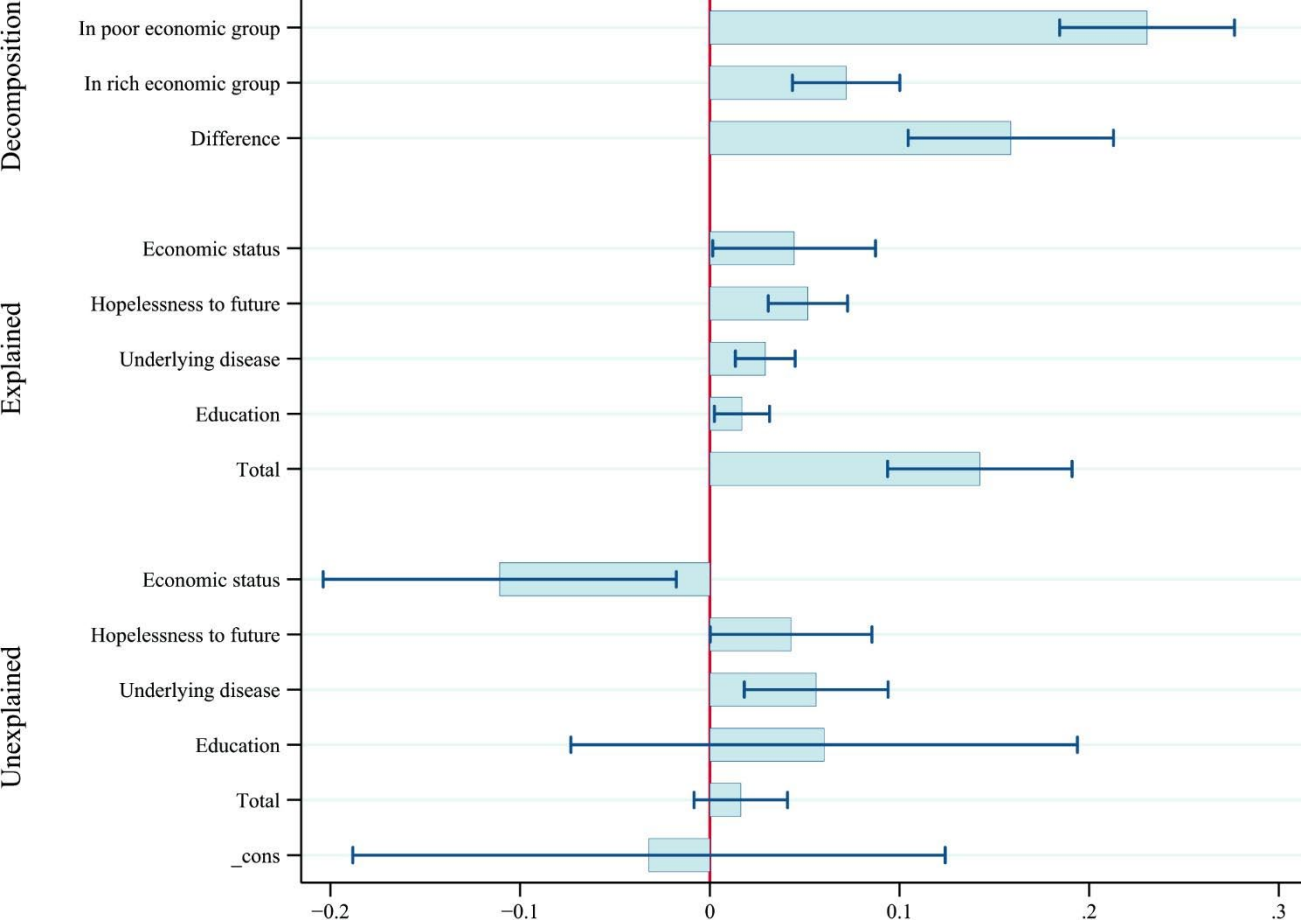
Result of Oaxaca Binder decomposition separated by overall, explained and unexplained portion and effect of determinants on inequality

In the unexplained portion, economic status (coefficient: -11.09; p: 0.020), hopelessness to future (coefficient: 4.26; p: 0.050) and underlying disease (coefficient: -5.63; p: 0.004) had significant effects. Figure 3 presents the results of inequality and its decomposition according to each variable.

## Discussion

Our data showed that less than 9% of participants assessed their health status as poor but there was a meaningful difference among different groups of SES in terms of SRH and the poor assessed their health lower than better-off people. In the study by Nejat in Tehran, almost the same proportion of people, 11.5%, assessed their health as bad and very bad [27]. This figure in the study in the USA by Kino [45] was 21%. These figures seem to be reasonable and people assessing their health as bad are lower than those with middle or good health status. In comparison with people from lower socioeconomic groups, being at the middle socioeconomic class or rich group reduces the chance of stating poor health by 0.44 and 0.62 respectively. Similar findings have been revealed by other studies showing socioeconomic gradients for SRH [46–49]. In the study by Mahvavi gorabi et al., they found a linear increasing pattern between SRH and different quintiles of SES [32]. Another study in six European countries found a strong relationship between SRH and several SES indicators including subjective social status and family influence [50]. According to the study by Nejat in Tehran, the chance of stating bad SRH in people in the poorest quintile was 4.3 times more than the richest [27]. Similarly, Kino and et al. [45] used education and household income as socioeconomic status indicators to examine to what extent socioeconomic disparities in self-rated health, hypertension, cardiovascular, and diabetes can be explained by health practices including avoiding smoking, drinking in moderation, healthy diet, regular physical activity, and adequate sleep. In their study they found that 0.39% of those with household income below federal poverty line reported their health as poor, in contrast this was 0.2 in their counterparts above the poverty line.

Hopelessness to future and suffering from an underlying disease were among the factors that had very negative effect of the status of SRH. Different things related to the private life of people and their family may cause people lose their hope for the future. It can also be influenced by the events happening around them which are out of their control. For example, over the last decade economic situation has been experienced a bad condition in Iran due to international severe economic sanctions and internal managerial challenges within the country. According to the findings, 20% of participants stated the history of losing their job which is high. Increasingly worsening economic situation in the country might be one of the root causes of not being hopeful for the future. Other studies have revealed a strong association between SRH and suffering from chronic diseases. For example in a study from Iran, it was shown that poorer SRH was significantly related to more chronic or long-term illness (OR, 1.61), greater psychological health disorders (OR, 1.69), and more dermatologic disorders (OR, 1.30) [33].

Also those with lower levels of education showed poorer status of self-rated health. In a study in India in 2014, health education explain 43.7% of SES-related inequalities in poor SRH among older adults [24]. In another similar study on older people in Hong Kong, findings confirmed that compare to those with the highest education attainment, those who had the lowest education attainment stated a higher risk of reporting poor SRH (RR = 1.77) [51]. Similarly in the study of Nejat in Tehran the odds of assessing SRH as bad in people with no formal education was 10 times more than those with tertiary education [27]. In another study done by Kino in USA, only 0.16% of people with high school graduate or above assessed their health as poor while this figure was 0.4 in individuals with less education [45].

It’s worth to mention that although other studies [46] shows that employment status and having health insurance coverage are associated with SRH, in the current study no relationship was found between SRH and these variables. Like the findings of Nejat study [27], no association was found in this study too among gender and SRH but regarding age, while the probability of having poor SRH increased with increasing age after controlling for other explanatory factors, in this study age did not impact SRH. Smoking, marital status, losing family members, history of mental health disorders or losing job, did not show statistically significant association with SRH.

The results of the Oaxaca-Blinder decomposition revealed a considerable gap in the poor status of SRH between the rich and the poor. The difference was 15.87% disfavoring the poor (p < 0.001). This difference was 14.4% in the study of Nejat in Tehran [27], and 16% in the study of Kino in USA [45]. In another study by Allen in Wales using the Oaxaca-Blinder decomposition 26.8% of survey respondents not being able to make savings reported being in fair/poor health, while it was 15.3% in respondents who were able to make savings, and the a difference between them was 11.5% points [52]. A large proportion (89.66%) of this difference was described by explained portion of the model; in other words, variables included in the model explained the gap between the poor and the rich to great extent. In the study of Kino in USA [45], the share of explained section of the Oaxaca-Blinder decomposition, comparing low and high income groups was 65%. This figure was 45.5% in the study of Allen in Wales comparing poor health between those who are able to make a saving of at least £10/month, and those who are not able using the Blinder-Oaxaca methodology [52].

The results of inequality decomposition showed that economic status was directly responsible for explaining 27.98% of overall inequality gap between rich and poor people. Moreover, hopelessness to the future, having an underlying disease and difference in the education level accounted for 32.64%, 18.34% and 10.71% of overall gap respectively. In a study by Yiengprugsawan et al. in Thailand, they showed that nearly half (47%) of the inequality in SRH was due to SES, particularly income state (approximately 28% of the contribution). Other main contributors were demographic features (31%) and region (21%) [53]. In the study of Nejat in Tehran the main contributors defining the estimated socioeconomic inequality were economic status (47.8%), level of education (29.2%) and age (23%), while gender and marital status had no contribution to the inequality in SRH [27]. The main contributors in the study of Kino in USA explaining the socioeconomic inequality were Marital status, Education and Smoking [45]. In the study of Allen in Wales, the Oaxaca-Blinder decomposition of the gap in prevalence of self-reported health between those who were able to make a saving of at least £10/month and those who were not, revealed that Social and Human Capital (26.4–40.4% of explained component) and Income Security and Social Protection (40.2–51.2% of explained component) were the main indicators responsible for the differences in fair/poor health [52].

### Study limitations and strengths

The current study was conducted in deprived regions, so it cannot be generalized to other parts of the country, including deprived regions. Additionally, the study only measured a small number of asset variables, which may not be enough to create a reliable socioeconomic status variable. This could lead to sampling bias. Despite these limitations, the findings of the study can still be used to address economic disparities in the field of SRH.

It is important to note that the observed inequality and the results of the decomposition do not necessarily indicate a causal relationship between the variables. The Oaxaca-Blinder decomposition methodology is a deterministic technique that decomposes a gap according to the factors included in the model. It is not able to ascertain the contribution of other variables. Although the Oaxaca-Blinder decomposition methodology is a useful tool for understanding the factors that contribute to inequality; however, it is important to note that this methodology does not prove a causal relationship between the variables. The methodology only decomposes the gap according to the factors included in the model, and it is not able to ascertain the contribution of other variables. In other words, the methodology can provide valuable insights into the factors that contribute to inequality, but it cannot be used to prove a causal relationship. The study has several strengths, including a large sample size, a high participation rate, and a population-based design. The study also adhered to methodology and quality control to reduce errors during data collection and analysis.

### Policy implications

Social justice is one of the most fundamental values mentioned repeatedly in the main upstream documents in Iran including Constitution of the Islamic Republic of Iran. Accordingly health equity has been emphasized on national health document such as 5-year national development plans. What health researchers can do is to investigate and provide a detailed and comprehensive picture about the current situation of health equity and the main contributors affecting it. These can force national and regional decision makers to understand and recognize the importance of health equity issues and take measures to tackle them. Therefore, it is necessary to have a comprehensive approach towards health equity. To do so, it is recommended other researchers do more researches on the subject and help shed light on other unknown aspects of inequity in health and try to find the main reasons behind it which makes it possible to pose more realistic and applicable policies accordingly. This also can help local and national health policy makers to assess the outcome of their proposed and implemented programs. According to the findings of our study, not being hopeful can affect health to great extent and it impacts the poor more severely. This implies that it can also affect other aspect of lives directly and indirectly. So further studies are needed to find reasons behind hopelessness and recommend short and long term applicable solutions to bring hope back to society.

## Conclusion

This study revealed pro-rich inequalities in the status of self-rated health within a deprived region in Iran. According the findings, hopelessness to future and having an underlying disease and difference in the education level were the main contributors of the existing inequity in SRH. These causes are not easy to be dealt with, as they are affected by many other identified and unidentified reasons. Regarding people suffering from underlying diseases, it is suggested to devise policies to improve access to/and remove healthcare utilization barriers for people suffering from underlying disease. To address hopelessness to future, it is recommended to carry out further studies to reveal factors which affect it in more details. This can help policy makers formulate more realistic and evidence-informed policies in order to lessen the current socioeconomic inequity in SRH. Local health officials should convince those decision makers responsible for allocating and distributing provincial budgets to allocate financial and in kind resources in a way to reduce disparity among people or provide more support for those from lower socioeconomic status and suffering chronic diseases. Taking into account the socioeconomic contributors of health inequity and shifting and targeting resources accordingly can lead to a more equal society and bring more hope for the poor.

### Electronic supplementary material

Below is the link to the electronic supplementary material.

Supplementary Material 1

## Data Availability

The dataset supporting the conclusions of this article is available upon editor request to corresponding author (Reza Pakzad, Email: rezapakzad2010@yahoo.com).

## Abbreviations

SES: Socioeconomic status
PCA: Principle Component Analysis
SRH: Self-rated health

## References

[1] Wachtler B, Hoebel J, Lampert T (2019). Trends in socioeconomic inequalities in self-rated health in Germany: a time-trend analysis of repeated cross-sectional health surveys between 2003 and 2012. BMJ open.

[2] Marmot M, Friel S, Bell R, Houweling TA, Taylor S (2008). Closing the gap in a generation: health equity through action on the social determinants of health. The Lancet.

[3] Marmot M, Allen J, Bell R, Bloomer E, Goldblatt P (2012). WHO European review of social determinants of health and the health divide. The Lancet.

[4] Arcaya MC, Arcaya AL, Subramanian SV (2015). Inequalities in health: definitions, concepts, and theories. Glob Health Action.

[5] Nourmoradi H, Kazembeigi F, Kakaei H, Jalilian M, Mirzaei A (2020). Assessment of knowledge, attitude, and practice toward covid-19 among a sample of Iranian general population. Open Access Maced J Med Sci.

[6] Armandpishe S, Pakzad R, Jandaghian-Bidgoli M, Abdi F, Sardashti M, Soltaniha K. Investigating factors affecting the prevalence of stress, anxiety and depression among citizens of Karaj city: a population-based cross-sectional study. Heliyon. 2023. 10.1016/j.heliyon.2023.e16901 PMC10279824 37346360

[7] World Health Organization. Handbook on health inequality monitoring: with a special focus on low-and middle-income countries, 2013. Geneva, Switzerland.

[8] Krieger N, Williams DR, Moss NE (1997). Measuring social class in US public health research: concepts, methodologies, and guidelines. Annu Rev Public Health.

[9] Cutler DM, Lleras-Muney A, Vogl T (2008). AdrianaSocioeconomic Status and Health: dimensions and mechanisms.

[10] Hashemi H, Pakzad R, Khabazkhoob M (2022). Decomposition of economic inequality in cataract Surgery using Oaxaca blinder decomposition: Tehran geriatric eye study (TGES). Ophthalmic Epidemiol.

[11] Hashemi H, Pakzad R, Yekta A, Aghamirsalim M, Ostadimoghaddam H, Khabazkhoob M (2021). Investigation of economic inequality in eye care services utilization and its determinants in rural regions using the oaxaca–blinder decomposition approach. Semin Ophthalmol.

[12] Rezaei S, Hajizadeh M, Irandoost SF, Salimi Y (2019). Socioeconomic inequality in dental care utilization in Iran: a decomposition approach. Int J Equity Health.

[13] Najafi F, Soltani S, Karami Matin B, Kazemi Karyani A, Rezaei S, Soofi M (2020). Socioeconomic-related inequalities in overweight and obesity: findings from the PERSIAN cohort study. BMC Public Health.

[14] Tissue T (1972). Another look at self-rated health among the elderly. J Gerontol.

[15] Jylhä M (2009). What is self-rated health and why does it predict mortality? Towards a unified conceptual model. Soc Sci Med.

[16] Garrity TF, Somes GW, Marx MB (1978). Factors influencing self-assessment of health. Soc Sci Med.

[17] Singer E, Garfinkel R, Cohen SM, Srole L (1976). Mortality and mental health: evidence from the Midtown Manhattan Restudy. Soc Sci Med.

[18] Mossey JM, Shapiro E (1982). Self-rated health: a predictor of mortality among the elderly. Am J Public Health.

[19] Crossley TF, Kennedy S (2002). The reliability of self-assessed health status. J Health Econ.

[20] Jürges H, Avendano M, Mackenbach JP (2008). Are different measures of self-rated health comparable? An assessment in five European countries. Eur J Epidemiol.

[21] Bombak AE (2013). Self-rated health and public health: a critical perspective. Front Public Health.

[22] Mansyur C, Amick BC, Harrist RB, Franzini L (2008). Social capital, income inequality, and self-rated health in 45 countries. Soc Sci Med.

[23] Inglehart Ronald B, Miguel D-M, Jaime H, Loek L, Ruud (2000). World values surveys and European values surveys, 1981–1984, 1990–1993, and 1995–1997.

[24] Srivastava S, Chauhan S, Patel R (2021). Socio-economic inequalities in the prevalence of poor self-rated health among older adults in India from 2004 to 2014: a decomposition analysis. Ageing Int.

[25] Elgar FJ, Pförtner T-K, Moor I, De Clercq B, Stevens GW, Currie C (2015). Socioeconomic inequalities in adolescent health 2002–2010: a time-series analysis of 34 countries participating in the Health Behaviour in School-aged children study. The Lancet.

[26] Elgar FJ, McKinnon B, Torsheim T, Schnohr CW, Mazur J, Cavallo F, Currie C (2016). Patterns of socioeconomic inequality in adolescent health differ according to the measure of socioeconomic position. Soc Indic Res.

[27] Nedjat S, Hosseinpoor AR, Forouzanfar MH, Golestan B, Majdzadeh R (2012). Decomposing socioeconomic inequality in self-rated health in Tehran. J Epidemiol Community Health.

[28] Machón M, Vergara I, Dorronsoro M, Vrotsou K, Larrañaga I (2016). Self-perceived health in functionally Independent older people: associated factors. BMC Geriatr.

[29] Yu LC, Zhang AY, Draper P, Kassab C, Miles T (1997). Cultural correlates of self perceived health status among Chinese elderly. J Cross Cult Gerontol.

[30] Torsheim T, Currie C, Boyce W, Kalnins I, Overpeck M, Haugland S (2004). Material deprivation and self-rated health: a multilevel study of adolescents from 22 European and north American countries. Soc Sci Med.

[31] Ahadi Z, Qorbani M, Kelishadi R, Ardalan G, Taslimi M, Mahmoudarabi M (2014). Regional disparities in psychiatric distress, violent behavior, and life satisfaction in Iranian adolescents: the CASPIAN-III study. J Dev Behav Pediatr.

[32] Gorabi AM, Heshmat R, Farid M, Motamed-Gorji N, Motlagh ME, Zavareh NH-T, et al. Economic inequality in life satisfaction and self-perceived health in Iranian children and adolescents: the CASPIAN IV study. Int J Prev Med. 2019. 10. 10.4103/ijpvm.IJPVM_508_17 PMC6547786 31198505

[33] Maharlouei N, Akbari M, Shirazy MK, Yazdanpanah D, Lankarani K (2016). Factors associated with self-rated health status in Southwestern Iran: a population-based study. Public Health.

[34] Najafi F, Rezaei S, Hajizadeh M, Soofi M, Salimi Y, Kazemi Karyani A (2020). Decomposing socioeconomic inequality in dental caries in Iran: cross-sectional results from the PERSIAN cohort study. Arch Public Health.

[35] Rezaei S, Ahmadi S, Mohamadi-Bolbanabad A, Khanijahani A (2020). Exploring socioeconomic inequalities in the use of medicinal herbs among Iranian households: evidence from a national cross-sectional survey. BMC Complement med ther.

[36] Zarabi A, Shahivandi A (2010). An analysis of distribution of economic development indices in Iran provinces. Geogr Environ Plan.

[37] Kakaei H, Maleki F, Biderafsh A, Valizadeh R, Mansournia MA, Pakzad I, Pakzad R. High prevalence of mental disorders: a population-based cross-sectional study in the city of Ilam, Iran. Front Psychiatry. 2023, 14. 10.3389/fpsyt.2023.1166692 PMC10293629 37383610

[38] Mohammed SH, Muhammad F, Pakzad R, Alizadeh S (2019). Socioeconomic inequality in stunting among under-5 children in Ethiopia: a decomposition analysis. BMC Res Notes.

[39] Yekta A, Hashemi H, Pakzad R, Aghamirsalim M, Ostadimoghaddam H, Doostdar A (2020). Economic Inequality in Unmet Refractive Error need in Deprived Rural Population of Iran. J Curr Ophthalmol.

[40] Zarei E, Pakzad R, Yekta A, Amini M, Sardari S, Khabazkhoob M (2021). Economic inequality in visual impairment: a study in Deprived Rural Population of Iran. J Curr Ophthalmol.

[41] Blinder AS. Wage discrimination: reduced form and structural estimates. J Hum Resour. 1973: 436–55.

[42] Oaxaca R (1973). Male-female wage differentials in urban labor markets. Int Econ Rev.

[43] Yun M-S (2004). Decomposing differences in the first moment. Econ Lett.

[44] Jann B (2008). A Stata implementation of the Blinder-Oaxaca decomposition. Stata J.

[45] Kino S, Kawachi I (2020). How much do preventive health behaviors explain education-and income-related inequalities in health? Results of Oaxaca–Blinder decomposition analysis. Ann Epidemiol.

[46] Gu H, Kou Y, You H, Xu X, Yang N, Liu J (2019). Measurement and decomposition of income-related inequality in self-rated health among the elderly in China. Int J Equity Health.

[47] Prus SG (2011). Comparing social determinants of self-rated health across the United States and Canada. Soc Sci Med.

[48] Alexopoulos EC, Geitona M (2009). Self-rated health: inequalities and potential determinants. Int J Environ Res Public Health.

[49] Woo J, Lynn H, Leung J, Wong S (2008). Self-perceived social status and health in older Hong Kong Chinese women compared with men. Womens Health.

[50] Moor I, Kuipers MA, Lorant V, Pförtner T-K, Kinnunen JM, Rathmann K (2019). Inequalities in adolescent self-rated health and Smoking in Europe: comparing different indicators of socioeconomic status. J Epidemiol Community Health.

[51] Lai ET, Yu R, Woo J (2021). Social gradient of self-rated health in older people—the moderating/mediating role of sense of community. Age Ageing.

[52] Allen J, Cotter-Roberts A, Darlington O, Dyakova M, Masters R, Munford L. Understanding health inequalities in Wales using the Blinder-Oaxaca decomposition method. Front Public Health. 2022. 10. 10.3389/fpubh.2022.1056885 PMC9797964 36589980

[53] Yiengprugsawan V, Lim LL, Carmichael GA, Sidorenko A, Sleigh AC (2007). Measuring and decomposing inequity in self-reported morbidity and self-assessed health in Thailand. Int J Equity Health.

